# Process optimization with rule-learning explainable AI, and its application to MRI scanning

**DOI:** 10.1371/journal.pone.0358070

**Published:** 2026-09-16

**Authors:** Sean Hartmann, Andrew Sharp, Heather Johnston, Michael S. Gee, Susie Y. Huang, Mukesh G. Harisinghani, Jens Gühring, Wei-Ching Lo, Vibhas Deshpande, Oleg S. Pianykh

**Affiliations:** 1 Mass General Brigham Hospital, Harvard Medical School, Boston, United States of America; 2 Siemens Healthineers, Malvern, Pennsylvania, United States of America; DAV University, INDIA

## Abstract

**Purpose:**

High complexity of modern processes renders manual process optimization impossible. The main goal of our work was to formalize and to demonstrate the practical efficiency of explainable rule-learning AI in complex process optimization.

**Materials and methods:**

Process optimization methodology was based on globally-optimal Boolean rule-learning AI models, capable of identifying multiple inefficiency patterns, and supporting process-oriented optimization metrics. To illustrate this approach in real-word environment, the study used 3994 liver and prostate Magnetic Resonance Imaging (MRI) exams performed on five 3T scanners at three outpatient facilities from January 2019 to January 2024. Imaging exams longer than 25 minutes were labeled as requiring optimization, which applied to 35.4% of liver and 52.6% of prostate cases. Rule-learning AI was applied to the MRI scanner log data to discover short and interpretable process optimization rules. The selected Boolean rules were used to implement improved exam protocols, and a permutation test was used to measure the statistical significance of the resulting change in average exam duration.

**Results:**

N=1000 top rules, identifying the most significant processing delay patterns, were discovered by rule-learning AI from the scanner log data based on F1 score. A smaller set of 20 top rules was selected using the secondary operational impact metric, an estimate of each rule’s impact on average scan duration. A change in liver MRI protocols based on findings from the top rule resulted in a 10.9% reduction of median scan time. The proportion of long liver exams was reduced from 35.4% to 23.9% (p < 0.001). Similar optimizations in prostate protocols were used to add a new scanning sequence, improving exam quality.

**Conclusions:**

Globally optimal, multi-model rule-learning AI can transform feature-rich device logs into concise, interpretable, and operationally meaningful rules. When combined with domain expertise, this approach can support measurable and sustainable improvements in complex clinical workflows.

## 1. Introduction

Modern workflows, driven by complex technology and narrowing resource constraints, are becoming increasingly challenging for human decision-making and optimization. Medical imaging (radiology) exemplifies this challenge: to optimize the duration of patient scanning, one must consider clinical needs, patient characteristics, types of imaging acquisitions, staffing, scanner settings, scheduling constraints, and more. This degree of optimization cannot be achieved manually, and cannot be based on hospital patient records alone, requiring more process-specific data and analyses [1,2].

Previous work has recognized the need for better process optimization algorithms and more detailed process data. On the methodological side, this has given rise to a new line of research exploring the use of classical *explainable AI* (XAI) methods, ranging from linear regression to rule lists, to uncover basic, human-interpretable processing logic [3–9]. On the applied side, automatically generated, feature-rich process log data has attracted growing attention for its potential to support human understanding of complex processes, including applications in medical imaging [10–13]. However, this work has just started – particularly in healthcare, where only a few recent studies have suggested using imaging scanner logs to extract more accurate measurements of patient movement, scanner utilization, and scheduling. Even in these studies, scanner log use was limited to serving as a source of exam and measurement sequence start-end times [2,14–17]. Consequently, the use of scanner logs in radiology has remained limited, largely due to the proprietary barriers in acquiring primary scanner log data, and difficulties in processing the large quantities of information contained in the logs.

As a result, leveraging the full set of device log features with explainable AI models to discover human-understandable process optimization rules still presents a compelling but unexplored opportunity for improving operational efficiency. Additionally, theoretical advances in this area must still be translated into real-world applications to demonstrate that XAI-derived insights can lead to tangible process improvements.

Motivated by this challenge in our daily clinical work, we set two principal goals of our study as follows: a) to develop practical criteria for using *rule-learning XAI* (RL-XAI) in complex process optimization, and b) to demonstrate how this methodology can lead to successful process optimization when RL-XAI is applied to complex device log data. Beyond achieving specific optimization gains, we also sought to better understand the mechanisms through which rule-learning XAI supports process improvement.

## 2. Methods

Our project consisted of two parts: first, we applied RL-XAI models to identify the most significant and interpretable patterns of processing delays, and second, we implemented model findings to improve scan efficiency, and to verify results in a real-world clinical setting.

This retrospective study was conducted in a large academic medical center. The historical data collected for this study was strictly operational, therefore ethical review and approval were not required, and the study was exempt by our institution from IRB oversight in accordance with applicable regulations; the exemption was granted under protocol 2022P002693. The data was accessed for research purposes from 10/01/2022–12/10/2025. Two paper authors had industry affiliations, but were involved only in providing and interpreting the scanner log data, and did not impact study model development, analyses, or conclusions.

### 2.1. Modeling criteria

Aiming at practical gains, we formulated the following criteria as the most essential for translating the XAI results into tangible process improvements:

1. *Globally-optimal solutions*: Finding the very best model(s), subject to a fixed number of features. When optimizing complex processes with many interconnected variables, it is critical to identify the most significant patterns of processing inefficiencies. Classical XAI algorithms, relying on faster greedy methods, do not guarantee finding the best solutions, which may result in inaccurate process interpretations.
2. *Multi-model optimization*: Finding the top *N* best models rather than a single model. For many problems, disparate explanatory models may provide similarly accurate predictions, but some may offer more actionable optimization targets than others. Most current XAI methods, including ensemble approaches, are designed to produce only a single final model.
3. Process-driven evaluation metric: The metric used to determine the best model should be adaptable to the problem in question. Conventional loss functions and metrics, traditionally used to optimize AI models, may not be useful or intuitive for process management. Supporting process-specific metrics extends model optimization to a broader range of real-world problems.

Together, these criteria define a general rule-learning optimization methodology that can be applied across various processes, and distinguish our approach from prior work. To illustrate their practical value, we used Magnetic Resonance Imaging (MRI) scan optimization, as one of the most complex medical imaging workflows, characterized by long and highly variable examination times [1,16,18,19].

### 2.2 Operational data, target, and features

This project used detailed MRI scanner logs, providing a granular and feature-rich representation of the imaging processes for contrast-enhanced *liver* and *prostate* exams – two common MRI scan types, associated with especially long and highly variable scan times. The scanner logs contained precise timestamps for all events performed during imaging exams, including scanner table movements, acquisition types, and user interface interactions (Table 1).

**Table 1:** Properties of liver and prostate examinations used in process optimization analysis. Each exam may be defined by a set of selected imaging sequences and protocols. Each exam instance results in multiple scanner log events, including imaging measurements (events associated with MRI image acquisition), which are aggregated at the exam level to create variables for the RL-XAI models.

	Liver Exams	Prostate Exams
Exam Count	1,857	2,137
Distinct Scanner Protocols	195	65
Distinct Imaging Sequences	31	26
Total Imaging Measurement Count	92,702	89,479
Total Log Event Count	223,028	231,812

**Fig 1:**
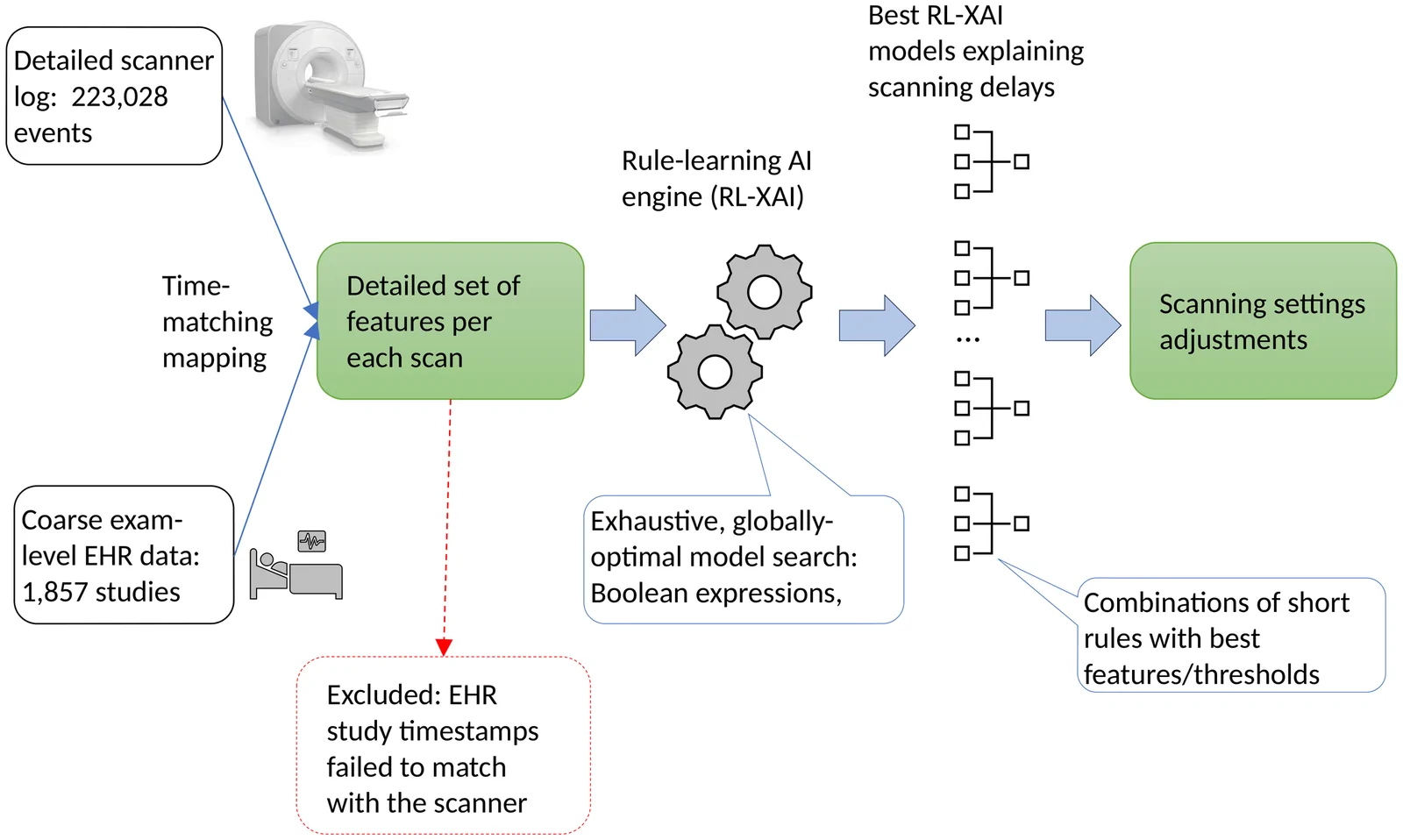
Study data and modeling flow. Scanner log data was joined with the EHR records, resulting in a large feature-rich dataset. This data was processed by the short RL-XAI engine, which identified the best most concise logical expressions, explaining scanning delays.

Because scanner logs record only device-specific information, we had to merge them with *Electronic Health Record* (EHR) data to establish clinical context, including examination and appointment-scheduling information. Since the two data sources shared no patient or examination identifiers, the scanner-to-EHR data mapping had to be done based on the scanner IDs and examination times, with each exam in the scanner log matched by timestamp to the nearest EHR exam performed on the same scanner. Not all exam records matched successfully: 28% of liver and 22% of prostate exams present in EHR were missing from the scanner logs, because the log data failed to save correctly during random time intervals due to networking issues. Additionally, 5.3% of exams labeled as abdominal in the scanner logs were not mapped to EHR records, because their scanner log timestamps did not overlap with EHR timestamps, reflecting random timing errors in either data source. In both cases, our investigation showed that missing records were uncorrelated with exam attributes. Therefore, the unmatched exams were excluded from our dataset, with no evidence that their exclusion introduced bias into our analysis (Fig 1).

The resulting log-to-EHR data mapping was instrumental for binding scanner log events to the overall scanning scheduling and efficiency. In particular, all patient MRI appointments at our facilities were scheduled for 30-minute slots, including 5 minutes of patient preparation time. Consequently, examinations with scan times exceeding 25 minutes left insufficient time to remove the patient from the scanner and begin the next examination on schedule. Liver and prostate MRIs were among the most frequent exam types exceeding the expected 25-minute scan limit and were thus selected as the focus of our study. The study included 1857 liver and 2137 prostate exams performed on five 3T MRI scanners from a major scanner vendor, at three outpatient facilities, from January 2019 to January 2024. 35.4% (239/676) of liver exams and 52.6% (631/1199) of prostate exams from January 2019 to September 2022, before any interventions were implemented, were too long for their scheduled time slots. 20% of exams from this period, stratified by class, were randomly assigned to the test set, and separate models were trained and evaluated for liver and prostate. Data from 1482 liver exams performed on three other MRI scanners at the same outpatient facilities that did not have their protocols optimized during the study period was used for comparison.

The mapping of device logs and EHR data also enabled us to collect and engineer 540 exam-level features, capturing as much process-related information as possible. Chosen in collaboration with the scanner engineering team, these features included counts of various MRI scanning events such as scanner table movements, coil changes, specific device alerts or processing failures; time-based information such as the day of the week and the number of exams performed on the scanner earlier that day; and exam-based information such as different exam types. The exam date was also included as a feature to identify whether differences in duration were more correlated with temporal trends than other exam properties. Each MRI scan included multiple imaging sequences (image acquisition steps). In order to reflect their interdependencies, we added not only their individual counts, but also groupings by two or three consecutive sequences, and their relative frequencies.

### 2.3 Model choice and feature extraction

The principal goal of this project was to use multi-model explainable AI (XAI) to achieve the maximum possible process time reduction; therefore, we chose the globally-optimal rule-learning XAI (RL-XAI) approach of “human knowledge models” (HKM) developed in [20]. As suggested by vast volume of cognitive research, humans can understand and apply decision models with at most four (Boolean) variables, where each variable is used at most once [21–23]. Therefore, the HKM method employs logic-based XAI learners that find the most accurate combinations of short Boolean rules, where both features and feature value thresholds are discovered to achieve the best explanation of a binary outcome from a complex multidimensional dataset.

This approach of combining short models with simple Boolean operators (“not”, “and”, and “or”) provides several key advantages essential for process optimization tasks. First, unlike conventional XAI approaches that produce numerical feature-importance rankings (SHAP, p-values, stepwise selection, model-specific importance algorithms), RL-XAI learns the best features together with the corresponding best rules, thereby capturing the best *sets of optimal features* with their actual optimal *predictive logic*. These simple and direct rules can be easily interpreted and evaluated by humans – compared to other XAI approaches such as regressions or Bayesian networks, which typically incorporate more complex sets of variables, math, and explanations. Second, while current feature importance techniques evaluate the contribution of individual features, RL-XAI is truly multivariate, identifying optimal combinations of features that operate jointly within a single model. Third, RL-XAI does not require feature preselection, which helps reduce concerns about multicollinearity: features included in the top RL-XAI models are unlikely to be redundant because these models outperform alternative models of the same size. Fourth, unlike conventional AI optimization which relies on suboptimal greedy algorithms, short RL-XAI learners enable *globally-optimal optimization* (computationally unfeasible for large models): considering all possible 2- or 3-feature subsets even from hundreds of original features may be done in a reasonable amount of time. Fifth, RL-XAI learners can accommodate application-specific loss functions, allowing optimization using the metric most relevant to the process being studied. Finally, exhaustive RL-XAI easily extends to a multi-model approach, producing the list of the first *N* best models instead of a single-model solution and thereby meeting our criteria for practical process optimization.

Interestingly, past research has shown that many complex systems may be governed by a very small subset of their parameters [24]. This justifies the use of small RL-XAI models, for which exhaustive (globally-optimal) model search is attainable despite having polynomial time complexity. Note that although many common statistical techniques, such as principal component analysis, can significantly reduce the original data dimensionality, they do not preserve the original set of variables [25]. In operational improvements, optimizing within the original feature set is imperative to identify the key process drivers that can be targeted with interventions to improve workflow efficiency.

### 2.4 Statistical methods

The second part of our study was to implement changes to protocols based on our findings from RL-XAI models. Changing scanner settings is a complex task which cannot be randomized in a busy outpatient clinic; therefore, we had to implement scanning protocol changes with a pre-post design.

In collaboration with clinical and engineering experts, and using the top RL-XAI models identified by our globally-optimal search, we selected the best “unbottlenecking” logical rules that could be implemented without sacrificing diagnostic quality. We then compared the difference in exam durations before and after these protocol changes.

During the time period of our analysis, scanner log data was available for 72% of liver exams and 78% of prostate exams performed at the institution, due to previously discussed networking errors. When evaluating the liver exam intervention, we analyzed data from 72% of a finite population of exams, so instead of conventional parametric statistical testing, we performed a two-sided two-sample permutation test to determine whether the reduction in average exam duration resulting from the intervention was statistically significant. The pre and post intervention group labels were reassigned 100,000 times using random permutations. The observed reduction in average exam durations was compared to the differences in average exam durations generated by these 100,000 permutations. Confidence intervals for model performance metrics on the train and test sets were computed using percentile bootstrapping with 10,000 samples. Statistical analysis was done using SciPy 1.10.1 (2023) and Python 3.8.19 (2024), and p-values below 0.05 were considered significant.

## 3 Results

The application of RL-XAI models to MRI scanner log data led to several important results identifying and eliminating major scanning inefficiencies, and illustrating the importance of the model selection criteria stated above.

### 3.1 Optimizing operational impact metric

The models selected as the best will depend on the choice of the optimization metric, and this choice should be operationally meaningful. Therefore, we used the following two metrics for evaluating each model’s quality on our MRI datasets:

- F1 score: a standard metric for measuring the quality of binary classification models in cases where classes may be imbalanced, and
- *Operational impact:* The product of the fraction of exams the model classifies as long and the average increase in duration among those exams.

Operational impact represents the average time savings achieved for the entire population of exams and can be formalized as an overall processing time gain provided by a given rule (model) m. Thus, if $n_{l}$and $\mu_{l}$ are the number and average duration, respectively, of exams that model m classifies as long, and $n_{s}$and $\mu_{s}$ are the number and average duration of exams predicted to be short, then overall operational impact $O_{imp}(m)\;$can be found as


$$\begin{array}{c} O_{imp}(m)=\left(\mu_{l}-\mu_{s}\right)*\;\frac{n_{l}}{n_{l}+n_{s}} \end{array}$$

For example, if a specific model identifies a logical condition of the exam (such as “exam contains at least 24 table movements”) which, if resolved, would shorten an exam’s duration by 5 minutes, but only 10% of the exams meet this condition (for example, have at least 24 table movements), the operational impact of this model will be 5*0.1 = 0.5 minutes. Thus, high operational impact helps workflow managers focus on the models leading to the most significant time savings overall. This metric is easily interpretable by operational teams, and its value directly relates to the practical implications of a given model.

**Fig 2:**
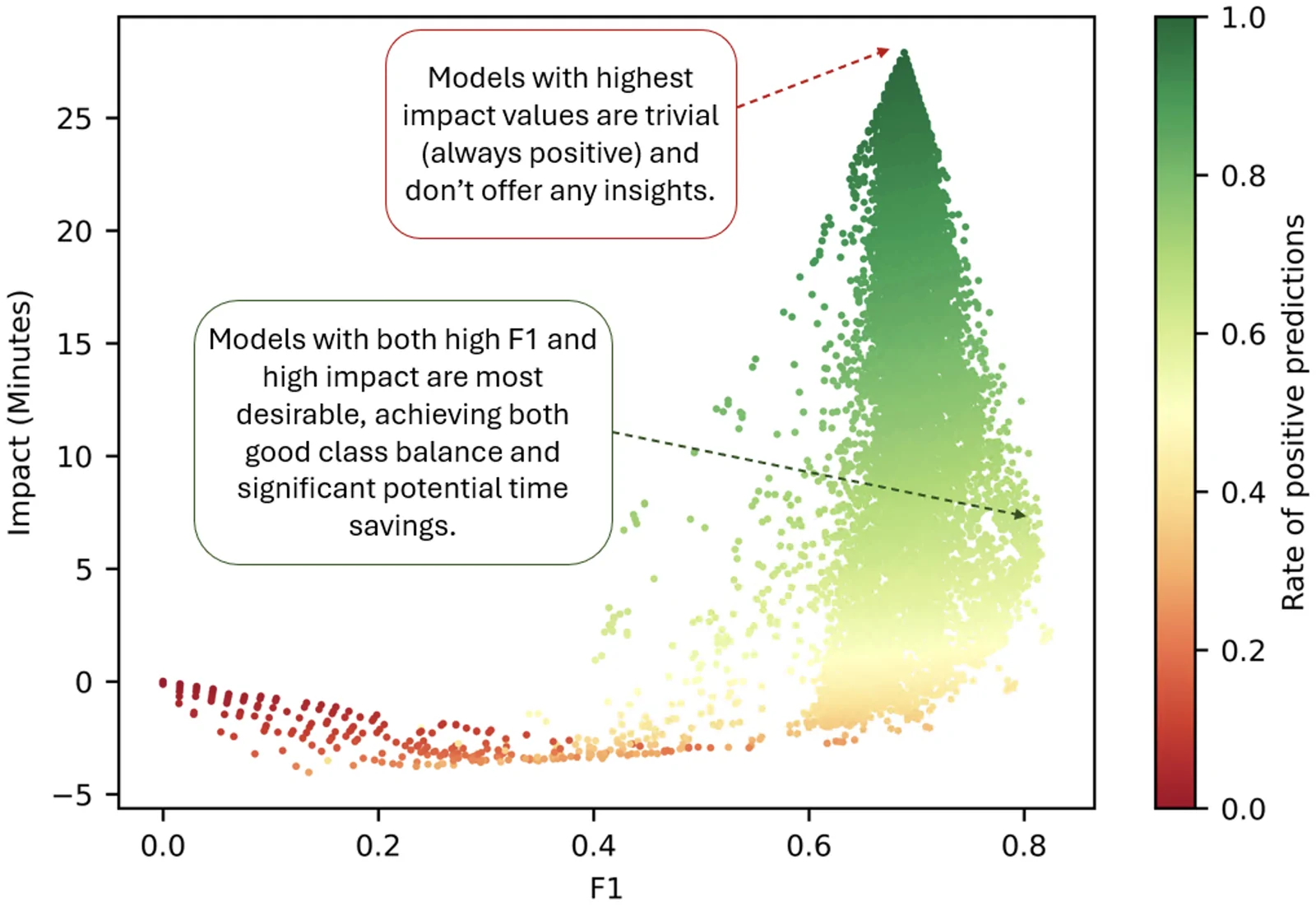
F1 and operational impact metrics for two-variable rules discovered by the RL-XAI models. Impact is defined as the product of frequency and average increase in duration. The highest-scoring rules differ depending on the metric used, and models performing well on both metrics are the most desirable. The color of each rule represents how closely its positivity rate matches the actual positivity rate of the dataset. The rules performing best based solely on the impact metric are imbalanced and almost always predict long exams.

Notably, both F1 and operational impact, if considered alone, would be insufficient as a sole optimization metric. For example, operational impact does not depend on the accuracy of the model and may be heavily influenced by a small number of samples. As seen in Fig 2, the models with the highest impact scores are those relying on imbalanced rules that identify very small groups of short exams, but the findings of these models likely cannot be applied to the broader exam population because their predictions are trivial (almost always positive). Although it may be useful in some operational settings to identify factors that are contributing to large outliers, we needed to identify rules that applied to a large number of exams, which operational impact alone cannot determine. F1, on the other hand, quantifies the ability of a given model to consistently distinguish between two classes, including in datasets where the classes are imbalanced and accuracy-based metrics may be poor indicators of predictive ability. However, F1 does not incorporate the magnitude of the difference between classes in any way. Models with high F1 scores may identify classes of exams that are consistently longer than other exams, but by only a few seconds, offering insignificant time savings in practice. The use of multiple metrics also provides a valuable way of comparing models that perform equally in the primary optimization metric. Using a secondary metric such as impact provides a way of deciding which model is preferable in this case and ensures Pareto optimality of the selected models.

This example demonstrates the importance of complementing the standard machine learning quality metrics with metrics that directly reflect the model’s operational value. If a model selection algorithm can find several high-quality models, then standard metrics such as F1 can be used for the primary optimization selection (in our case, to consistently separate long exams from short ones). Once we had produced a set of top rules based on F1, we then ordered them by operational impact to determine which workflow improvements could lead to the most potential time savings.

### 3.2 Learning the best rules

**Fig 3:**
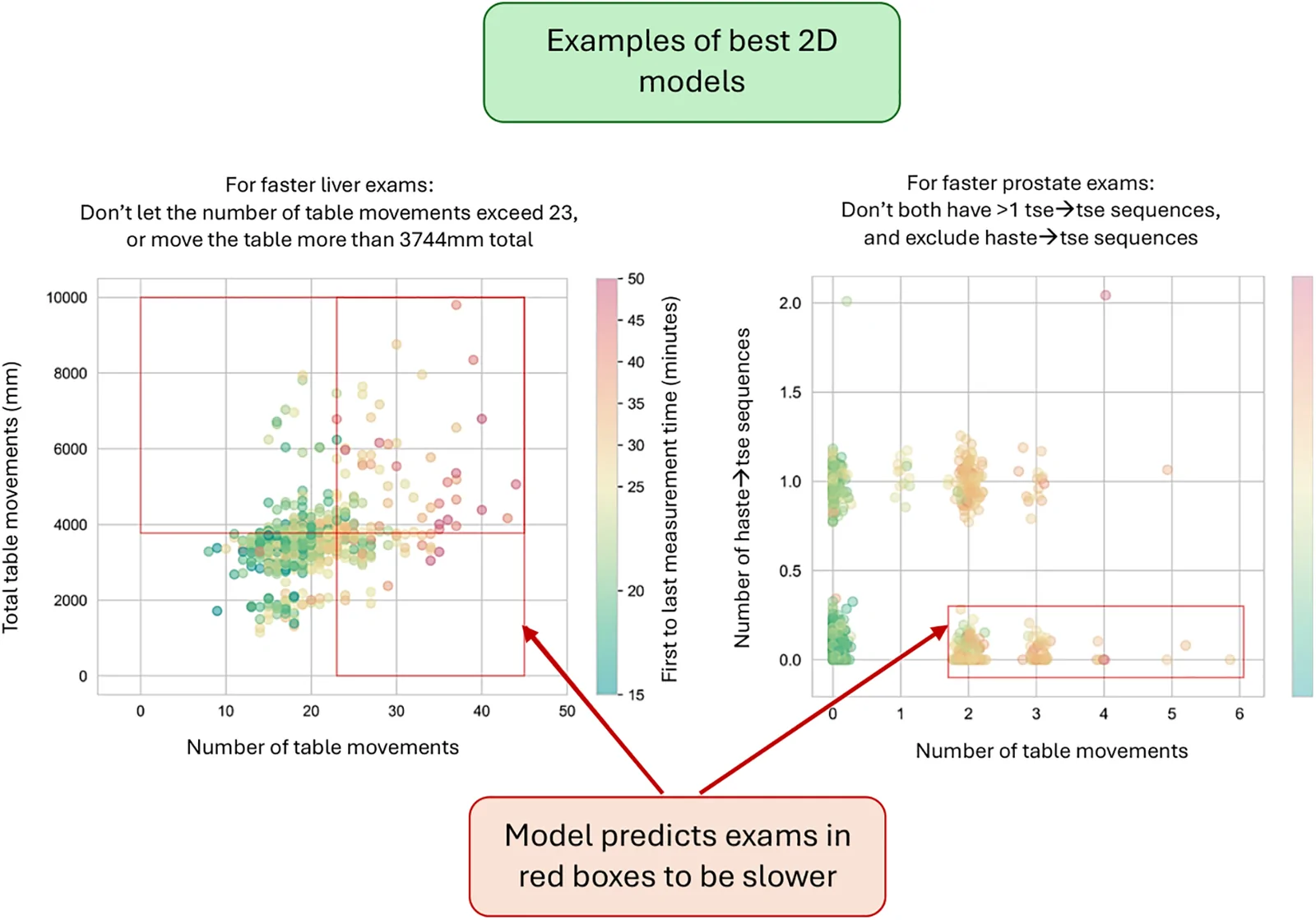
RL-XAI models found factors associated with longer gradient times, such as excessive table movements in liver exams. Each point represents a single exam instance; greener shading indicates shorter exams, and red indicates longer. Left: example of a best Boolean rule with OR, liver MRI exams; Right: example of a best Boolean rule with AND, prostate MRI exams.

Training the best 2-feature RL-XAI models took 30 minutes, and 3-feature – 1 day, on a plain, off-the-shelf PC (3.40 GHz CPU, 16.0 GB RAM). In each case, N = 1000 best models were found based on F1 score, and then the top 20 models were chosen based on operational impact. In addition to the two quality metrics, we visualized the relationships between each best model rule and the actual data, as shown in Fig 3.

A few top rules found by the RL-XAI models to be most predictive of long MRI exams are shown in Table 2. The use of both F1 and operational impact metrics ensured that the resulting rules were consistently associated with long exams and presented opportunities for significant time savings.

**Table 2:** Examples of best rules for predicting long liver (top) and prostate (bottom) MRI exams, with individual features highlighted. These rules were discovered by RL-XAI algorithms, which identified the optimal choices of variables and thresholds. Impact was defined as rule frequency multiplied by its average difference in duration – the impact on shortening the entire workflow. {} with arrows indicate specific acquisition subsequences contributing to longer scan times. Numbers in parentheses are 95% confidence intervals computed via 10,000 bootstrap samples. “3T Vida” refers to the specific scanner model, (shims, coils) – to scanner hardware settings, and (tse, haste, fl3d_vibe, ep2d_diff) and similar – to various scanning sequences performed by MRI scanner.

Rule for predicting long (> 25 min.) exams	F1, Train Set	F1, Test Set	Operational Impact, Train Set	Operational Impact, Test Set
**Liver MRI**				
Shims>=4 **AND** FreSeq adjustments>=8	0.62 (0.57-0.67)	0.59 (0.48-0.69)	1.9 (1.4–2.4) min	2.2 (1.0–3.4) min
Duplicated measurements>1 **AND** CoilSensSeq adjustments >=5	0.50 (0.43-0.56)	0.47 (0.32-0.60)	1.3 (0.9–1.7) min	1.9 (1.0–3.0) min
No {haste→fl3d_vibe→fl3d_vibe} sequence **OR** final measurement! = fl3d_vibe	0.68 (0.63-0.73)	0.67 (0.56-0.76)	2.3 (1.8–2.9) min	1.8 (0.8–2.9) min
Table movements>24 **OR** Shims>=4	0.62 (0.58-0.67)	0.60 (0.50-0.69)	2.3 (1.7–2.9) min	2.2 (0.7–3.7) min
Table movements>=24 **AND** Table movement direction changes>=7	0.45 (0.38-0.52)	0.49 (0.34-0.61)	1.4 (1.0–1.8) min	1.9 (0.9–2.9) min
Table movements>=24 **OR** table movement distance>=3775	0.54 (0.48-0.60)	0.51 (0.39-0.62)	1.6 (1.2–2.1) min	1.9 (0.8–3.0) min
**Prostate MRI**				
{tse→tse} sequences>=1 **OR** CoilSensSeq adjustments! = 6	0.79 (0.76-0.82)	0.78 (0.73-0.83)	3.8 (3.4–4.1) min	3.8 (3.1–4.6) min
tse t2_cor measurement>=1 **OR** CoilSensSeq adjustments!= 6	0.78 (0.75-0.81)	0.78 (0.73-0.83)	3.6 (3.2–4.0) min	3.8 (3.1–4.6) min
{haste→fl3d_vibe} sequence **OR** (Table movement direction changes>=3 **AND** Tse measurements>=2)	0.66 (0.63-0.69)	0.69 (0.63-0.74)	1.0 (0.4–1.5) min	0.7 (−0.7–1.9) min
3T Vida scanner **AND** ({tse→tse} sequence>=1 **OR** (No wip_990_BodyDiffusion_ep2d_diff measurements))	0.67 (0.63-0.70)	0.68 (0.62-0.74)	1.8 (1.4–2.2) min	1.6 (0.7–2.4) min
{fl3d_vibe→haste} sequence **AND** (No {haste→tse} sequence **OR** less than 50% of measurements are haste)	0.67 (0.64-0.70)	0.69 (0.63-0.74)	1.0 (0.4–1.6) min	0.8 (−0.9–2.1) min

### 3.3 Operationalizing RL-XAI results

**Fig 4:**
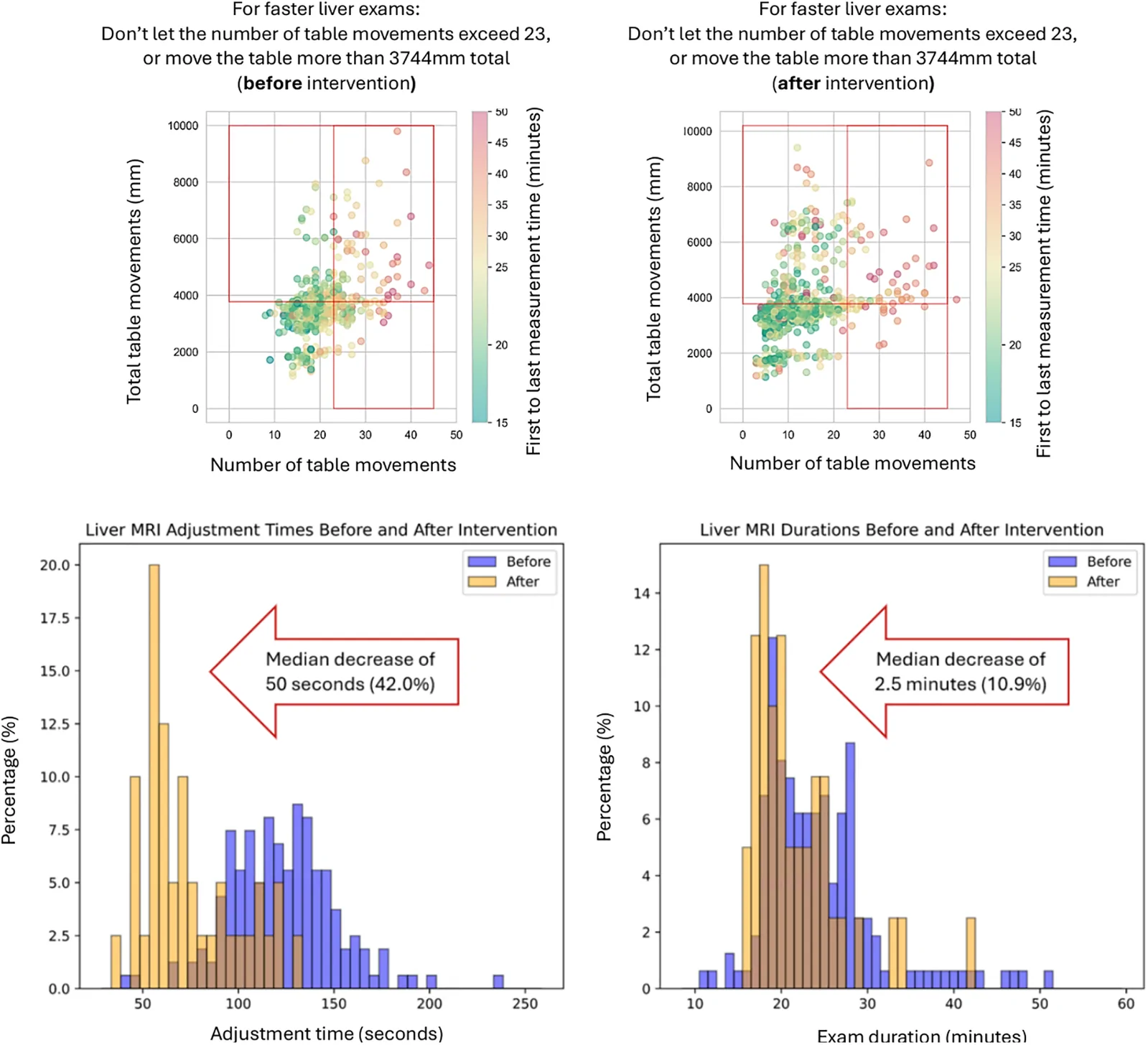
RL-XAI models driving operational improvements. Top left: Excessive table movements were identified as a major contributor to scanning bottlenecks. Top right: Reductions in table movements after the identified improvements were implemented in October of 2022. Bottom: Reductions in adjustment times and total exam durations after table movements were reduced.

The combined clinical and engineering team reviewed the top 20 models with the highest operational impact and best F1 scores, indicating sufficient class balance, to identify the features which could be changed without reducing the clinical value of the scan. Based on this review, the team implemented several protocol changes, prioritizing the most meaningful and operationally-impactful rules. These changes led to significant improvements in scan times, as shown in Fig 4.

For example. the team decided that number of scanner table movements was a good target for liver MRI improvement because there was no direct clinical need for the high number of table movements associated with longer exams, and because it was operationally feasible to implement a reduction in table movements separately from any other protocol changes, allowing for evaluation of the change in exam times. The existing protocols had been designed without specific consideration of the number of table movements since the table movement time was negligible. However, these movements were found to be triggering extra adjustments that were in some cases adding several minutes to the total exam time, even for table movements that were too small to be of significant clinical benefit. New protocols removing these too-small table movements were put into place at the outpatient facilities used for this study. Thus, by following only one of several discovered RL-XAI rules, we were able to decrease the median duration of liver exams by 2.5 minutes, a 10.9% reduction. The application of this single optimal rule reduced the proportion of long (> 25 minute) exams from 35.4% (239/676) to 23.9% (282/1181).

To determine whether this reduction in average liver MRI durations was statistically significant, we performed a permutation test with 100,000 random permutations and a significance threshold of 0.05. This test resulted in a p-value less than 0.001, so we concluded that the observed reduction in exam times was statistically significant. This conclusion was independently verified by comparison to the three other scanners, which did not have their protocols optimized, at the same outpatient facilities. On those scanners the median liver exam duration did not improve (24.5 minutes from January 2019-September 2022 versus 24.9 minutes from October 2022-January 2024). The proportion of long liver exams on these scanners was 47.7% (594/1245) in the pre-intervention period and 48.9% (116/237) in the post-intervention period, again showing no improvement on the non-optimized scanners.

Table 3 summarizes the results of the process optimization, guided by our RL-XAI models.

**Table 3:** Descriptive statistics of liver exam times before and after implementation of table movement reductions. Note the reduction in average and median exam times after implementation.

Date Range	January 2019-September 2022 (before intervention)	October 2022 – January 2024 (after intervention)
Exam count	676	1,181
Average exam time (minutes)	24.3	22.9
Standard Deviation (minutes)	6.4	6.7
Median exam time (minutes)	22.9	20.4
5^th^ percentile exam time (minutes)	18.8	16.9
95^th^ percentile exam time (minutes)	35.0	38.0

Note that despite the natural variability in patient imaging, our RL-XAI-guided improvement remained significant throughout the long post-intervention time range (October 2022 – January 2024), confirming a sustainable improvement. Both liver and prostate optimizations resulted in significant time savings – in liver this time was used to shorten the exams, while in prostate it was invested in improving protocol quality (including the addition of a large diffusion imaging sequence and other optimizations to image quality).

### 3.4 Globally-optimal, multi-model RL-XAI learning

**Fig 5:**
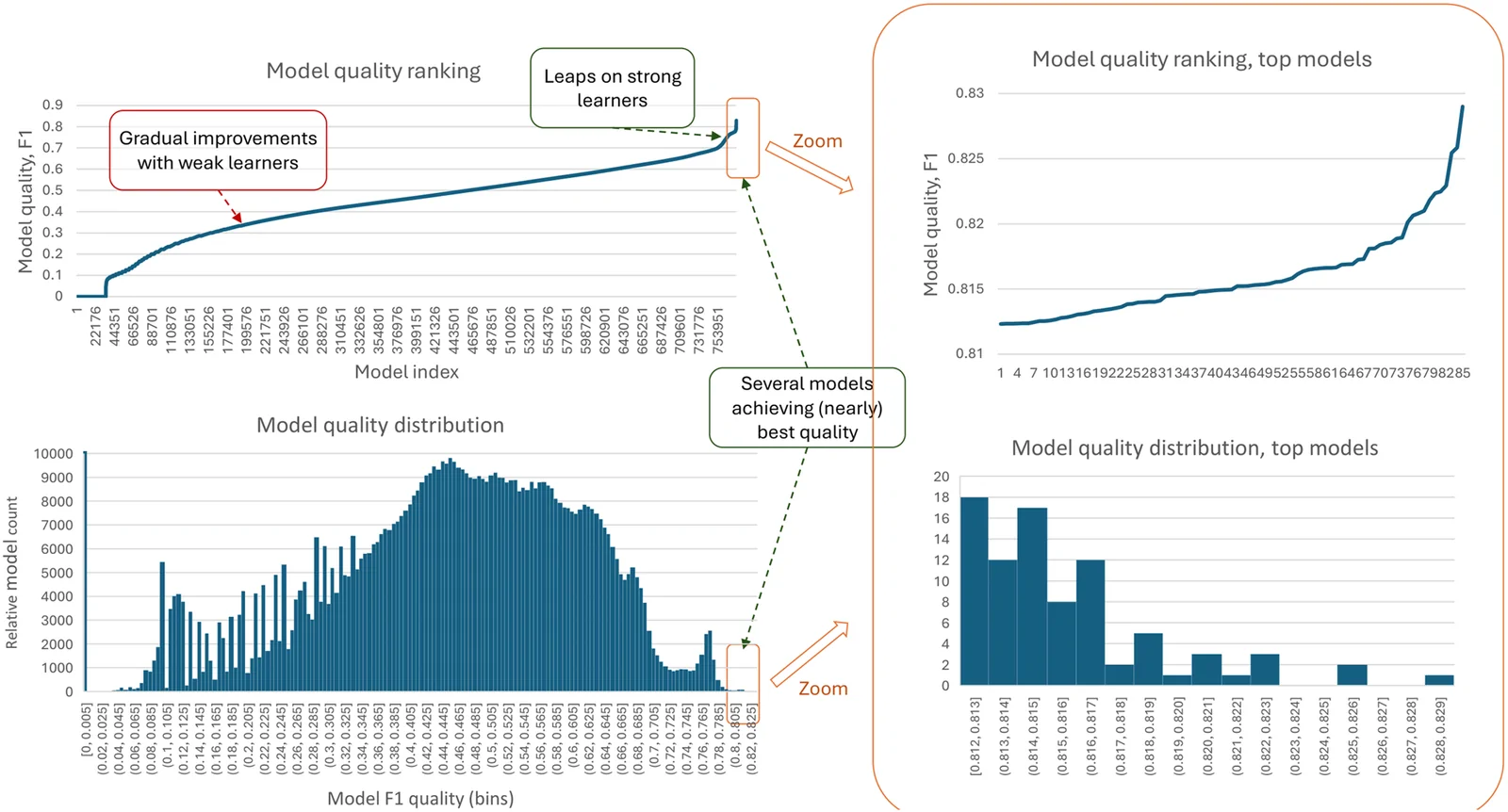
Model quality in multi-model RL-XAI learning, extracted from 800,000 RL-XAI models of three variables (using F1 quality metric). Note a sharp increase in the model quality, achieved by the few very best models.

Running multi-model RL-XAI optimization on a large set of MRI scanner log features offers interesting insights into the model learning process, presented in Fig 5.

The model quality (F1) ranking curve in Fig 5 shows the sorted F1 values across the range of all considered RL-XAI models, evaluated on the test data. While model quality follows an overall gradual and smooth pattern, there is a marked increase among the few highest-performing models on the far right. The quality histogram demonstrates that the vast majority of possible models have low quality performance, while only a tiny fraction achieves the highest quality values.

We found this pattern to be common among various modeling experiments, emphasizing the key role of the optimization criteria we stated earlier. *Globally-optimal* search is important, because the best models are exceptionally rare and may not be found with suboptimal techniques (such as greedy search algorithms) or under limited time. Finding *multiple optimal models* becomes equally vital, as high-quality models using different feature sets can provide alternative avenues for process improvement, especially in complex and evolving systems.

## 4 Discussion

Our study demonstrated the advantages of three principal criteria for XAI-driven process improvement introduced in this work, and revealed several important aspects of using RL-XAI in guiding operational decision-making.

First, applying explainable rule-learning models to feature-rich scanner log data enabled us to automatically sift through millions of small, interpretable “bottleneck explainers”, and identify the few with the greatest impact on scanning process duration. Doing this manually would be impossible; using traditional “feature importance” XAI would discover neither the rules of inefficiency, nor their specific subsets of features. In this respect, RL-XAI created a completely new opportunity to mine complex data, enabling human experts to understand the major sources of process inefficiencies. *In essence, our approach revealed an effective, new pattern of AI–human collaboration, where each side was doing what it does best: AI discovered the most predictive and interpretable patterns in the complex data, while humans translated those patterns into meaningful, actionable process-improvement interventions*.

Second, using globally-optimal search for *N* best models supported maximum reduction of the scanning time, while providing the opportunity to select the most practical and clinically acceptable options. As we demonstrated, the very best models can be significantly better than the models that are “good on average”, but they are also exceptionally rare (Fig 5). This level of exhaustive search became feasible only because RL-XAI models are compact and use fast Boolean mathematics – the same exact key properties that make these models highly interpretable. This combination yielded a very practical and transparent optimization approach.

Third, the explainability of the best RL-XAI rules and the use of the operational impact metric enabled our operational and clinical expert teams to understand the key reasons behind each rule, its practical impact on the operations, and its clinical value. As a result, this led to the most meaningful interventions. This has become particularly visible in our study, where operational optimization prevailed for the liver exams (saving time), while clinical optimization prevailed for the prostate scans (using time savings to add a new clinically important imaging sequence). Exploring the best RL-XAI rules with the process-specific operational impact metric helped us understand the actionable interpretation of each best model, and its most meaningful impact on our patient population. In this regard, it is equally important to understand that the best RL-XAI rules are not necessarily causal, and were not meant to be. While table movements were associated with prolonged exams, the most proximal cause was the lengthy adjustments triggered by unnecessary movements. Complex processing data, and healthcare data in particular, is often composed of myriads of confounding events, rendering automatic causality discovery challenging, if not impossible [26–29]. Therefore, domain knowledge and human involvement are imperative both to evaluate potential causal pathways, and to determine actionable insights. RL-XAI provided the foundation for the interaction between the domain experts and hidden processing logic.

Finally, although we illustrated our work with an application to optimizing medical image processing, the approach itself is universal, extending to any process. The key power of AI–human collaboration provided by our method can benefit many applied areas, guiding their operational management with explainable, high-quality optimization rules.

This study has natural limitations. First, device log data is often proprietary, and not always readily available or easily interpretable. Second, while our methodology is generalizable, device logs will remain vendor- and device-specific, limiting the choices of supported features and outcomes. Third, given the continuous nature of process optimization it may be challenging to separate the impact of implementing insights from RL-XAI models versus parallel and subsequent technological and process improvements. Finally, some processes may not be efficiently explained by small models, or may not be efficiently explainable at all (for instance, due to high randomness and variability). These considerations, as well as the feasibility of implementing any improvements based on the models’ findings, should be taken into account before planning for a similar project.

In conclusion, in practical settings where process complexity challenges human understanding, explainable and interpretable models extracted from process logs are essential for identifying the key drivers of process efficiency. By applying globally optimal, multi-model RL-XAI to feature-rich scanner-log data, one can identify concise, interpretable process-optimization rules that guide high-impact and operationally meaningful interventions.
